# The impact of genre-based instruction on Saudi university students’ English writing performance and motivation: a mixed-method study

**DOI:** 10.3389/fpsyg.2024.1387009

**Published:** 2024-05-24

**Authors:** Muhammad M. M. Abdel Latif, Talal Musaed Alghizzi, Tahani Munahi Alshahrani

**Affiliations:** ^1^Faculty of Graduate Studies of Education, Cairo University, Giza, Egypt; ^2^College of Languages and Translation, Imam Mohammad Ibn Saud Islamic University (IMISU), Riyadh, Saudi Arabia

**Keywords:** genre-based instruction, writing motivation, writing pedagogy, writing psychology, writing performance, genre-based writing instruction

## Abstract

Despite the important role motivation plays in students’ writing learning and development, not much attention has been given to experimenting particular instructional techniques for developing students’ writing motivation. One of the least researched teaching techniques in writing motivation studies is genre-based instruction. In this study, we examined the impact of genre-based instruction on 21 Saudi university students’ English argumentative and classification essay writing performance and their writing motivation dimensions (writing apprehension, anxiety, self-efficacy and self-concept). Drawing upon the quasi-experimental research design and non-random sampling technique, we used genre-based instruction with a representative intact class of English-as-a-foreign-language (EFL) writing students at a Saudi university. To examine the potential impact of the treatment, we collected pre- and post-instruction data, along with data gathered through an open-ended questionnaire. The results showed that genre-based instruction has contributed significantly to improving the students’ writing performance and also their writing motivation dimensions. These positive gains varied from one writing quality aspect and motivational variable to another. The students’ answers to the open-ended questionnaire also showed the positive writing learning and motivation improvements they experienced. It is generally concluded that developing students’ language and rhetorical awareness and text composition performance seems to be a prerequisite for improving their writing motivation levels. The paper ends with discussing the implications of the results.

## 1 Introduction

Learning and instructional practices play an important role in shaping students’ writing motivation. Research generally indicates that the larger portion of language and writing learning motivation factors concern instructional practices rather than learning ones (e.g., Atay and Kurt, 2006; Lo and Hyland, 2007). Despite this, examining the motivating impact of particular writing teaching techniques on students’ affect is an issue yet to be given due attention.

Previous instructional studies have examined the impact of interventional treatments on students’ writing performance and motivation. The treatments used in previous research include: technology-supported instruction (e.g., Li et al., 2014; Zhang et al., 2014; Jiang and Luk, 2016; Kramer and Kusurkar, 2017), strategy instruction (e.g., Limpo and Alves, 2014; De Smedt et al., 2019), feedback treatments (e.g., Duijnhouwer et al., 2012; Tang and Liu, 2017; Yao, 2019), and task interest-based instruction (e.g., Hidi and Anderson, 1992; Hidi et al., 2002). While there is some reasonable research on such interventional types, scarce studies have looked at how students’ writing performance and motivation could be fostered by genre-based instruction. There is a dire need for addressing this issue in some particular foreign language writing learning environments. For example, students in the Saudi university context experience writing skill deficiencies causing them to be demotivated to write (e.g., Abdel Latif, 2011; Altukruni, 2019; Qasem and Zayid, 2019). Helping these studies overcome their English writing performance problems and demotivation requires teaching writing to them using different instructional techniques (e.g., Al-Khairy, 2013; Khadawardi, 2022). Therefore, in this study we examined the potential impact of genre-based instruction on Saudi university students’ English writing performance and motivation.

## 2 Literature review

### 2.1 Writing motivation and its correlates

Motivation plays an essential role in students’ writing learning, performance and development. Writing motivation is generally conceptualized as a multifaceted construct which interacts with learners’ developmental stages, disciplines and environments (Wright et al., 2019). There have been some previous attempts in conceptualizing writing motivation (e.g., Hidi and Boscolo, 2007; Wright et al., 2019; Abdel Latif, 2021). In light of such taxonomies, learners’ motivation is viewed as an umbrella term encompassing their liking or disliking of writing situations and perceived value of writing, the situational feelings they experience while writing and the way they regulate them, the beliefs about their writing ability and skills, and their desired goals for learning to write (Abdel Latif, 2021). With this definition, writing motivation constructs can be categorized into four main types: (a) attitudinal/dispositional constructs (writing apprehension and perceived value of writing); (b) situational constructs (writing anxiety and motivational regulation of writing); (c) writing ability belief constructs (writing self-efficacy and self-concept); and (d) writing learning goal constructs such as writing achievement goal orientations (see Abdel Latif, 2021).

Literature indicates that students’ writing motivation is associated with some variables. Abdel Latif (2021) classifies these variables into the following three categories: (a) personal variables (i.e., age, gender and socio-cultural background); (b) performance and belief correlates such as language ability, writing performance, and perceived language and writing competence beliefs; and (c) learning and instruction practices which include the writing topics assigned, and the teaching materials and instructional techniques used. Research implies the motivating impact of assignment topics on students’ writing motivation (e.g., Behizadeh and Engelhard, 2014). Some studies also indicate that students’ writing demotivation can stem from inappropriate instruction practices (Atay and Kurt, 2006; Abdel Latif, 2015), or the lack of interesting teaching materials (Lo and Hyland, 2007). Accordingly, optimizing writing instruction is key to getting students motivated to write.

### 2.2 Previous research on genre-based writing instruction

Genre-based instruction is an explicit and consciousness-raising writing teaching technique designed for helping students be familiar with the communicative purposes and the linguistic structures and rhetorical conventions and features of particular genres (see Hyland, 2004, 2016). It draws mainly on getting students to model these features in their own texts. Such technique is particularly important to second language (L2) writing students as it could help in improving their linguistic and communicative abilities (Leki et al., 2008; Paltridge, 2013). It enables students to become successful members in their disciplines by familiarizing them with academic writing norms in the target genre, and with the language and genre features constituting its discourse (Van de Poel and Gasiorek, 2012).

Previous studies testing the impact of genre-based instruction on students’ writing are generally scarce. Some of these few studies examined how the use of genre-based feedback influences students’ text revisions. For example, Martínez Esteban and Roca de Larios (2010) engaged their L2 students in revising their written texts based on noticing and comparing them to native-speaker model ones. They found that model text noticing and comparison helped their students to incorporate a considerable number of textual and linguistic features in their subsequent revisions. Luquin and Mayo (2021) also examined the effect of collaborative genre-based revision on L2 students’ text quality, and ability to solve content and linguistic problems while revising texts. Their study revealed that the experimental group students were able to incorporate a significantly larger number of mechanics- and discourse-related aspects into the post-treatment writing. It is worth to note that these two studies did not deal with the impact of genre-based instruction on students’ writing motivation.

There is a paucity of research investigating the way students’ writing motivation may be influenced by genre-based instruction. Yasuda (2011) reported a study which mainly addressed how genre-based tasks foster L2 students’ language knowledge and writing performance. Her interview data showed that the participants’ improved language knowledge levels resulting from genre-based instruction led them to have subsequent better writing ability confidence. Two in-depth studies addressing the impact of genre-based instruction on writing motivation were reported by Van de Poel and Gasiorek (2012) and Han and Hiver (2018). Van de Poel and Gasiorek (2012) used genre-based instruction in two writing courses. Their data showed that the students significantly perceived themselves as more competent writers and had a more positive attitude toward getting feedback from teachers and peers. On the other hand, Han and Hiver (2018) investigated the influence of genre-based instruction upon L2 students’ writing self-efficacy and apprehension. They assessed these two motivational constructs using scales before and after instruction, and students’ reflective journal and interviews. Han and Hiver found that genre-based instruction positively impacted their students’ writing self-efficacy; such improvement was ascribed to the writing mastery experiences the students had during the genre-based instruction received. However, they found an increase in the students’ writing apprehension, which was attributed to their previous poor writing experiences. Apart from its motivating impact, the two studies published by Van de Poel and Gasiorek (2012) and Han and Hiver (2018) also found a positive effect for genre-based instruction on students’ writing performance.

Due to the scarcity of relevant research and also the narrow scope of these previous few relevant studies, there is a need for further research exploring the relationship of genre-based instruction with students’ writing motivation from a broader angle. First, we can combine writing performance with writing motivation data; such combination will show us how any potential improvement in students’ writing performance relates to their writing motivation. Second, it can be noted that the previous few more relevant studies (e.g., Van de Poel and Gasiorek, 2012; Han and Hiver, 2018) only dealt with students’ writing self-ability beliefs and apprehension. Therefore, there is a need for addressing a wider range of writing motivation constructs in genre-based instruction research. Finally, it is also important to look at the effect of genre-based instruction on students’ performance on two task types; this will give us a clearer picture about its potential impact. Tackling these issues in the Saudi university context could help students overcome the writing performance and motivational problems revealed in previous research (e.g., Abdel Latif, 2011; Al-Khairy, 2013; Altukruni, 2019; Qasem and Zayid, 2019; Khadawardi, 2022).

## 3 The present study

The present study tried to address these gaps by looking at the effect of genre-based instruction on Saudi university students’ English writing performance on two task types, and on their writing apprehension, anxiety, self-efficacy and self-concept. Thus, the originality of the present study stems mainly from addressing these four writing motivation constructs rather than one or two motivational variables only. Accordingly, the present study is guided by the following three research questions:

- To what extent does genre-based instruction influence Saudi university students’ English writing performance on argumentative and classification tasks?
- How does genre-based instruction affect Saudi university students’ English writing apprehension, anxiety, self-efficacy and self-concept?
- How do Saudi university students perceive genre-based writing instruction and its potential impact on their writing motivation?

As may be concluded, the present study depended on the quasi-experimental research design. We specifically used the one-group pre-post-test design which allows a deeper observation level for comparing the participants’ scores on the same measures before and after the treatment (Johnson and Christensen, 2019). We experimented genre-based writing instruction with a sample of English majors at a Saudi university. The study made use of a mix-method research approach by collecting pre- and post-test data, along with a post-course evaluation questionnaire. With this research approach, the study could have important contributions to writing instruction and motivation literature.

### 3.1 Participants

The study was conducted at a Saudi university with a sample of students majored in English. The sample consisted of 21 students who were in their second year of university study. The students were all in one intact class, and they were attending a 4-year English language teacher/translator education program during the data collection stage. During the data collection stage, this class included the highest number of English writing students in the target university campus. The study drew upon convenience sampling which is a non-random sampling technique. Despite its limitations, convenience sampling is the most commonly used sampling technique in L2 teaching studies (Farrokhi and Mahmoudi-Hamidabad, 2012). In light of literature-based recommendations (e.g., Mackey and Gass, 2005; Dörnyei, 2007), the potential bias in this sampling technique was overcome by selecting a highly representative intact class of English writing students at this level. The students in this intact class resembled second-year peers in their previous university English writing experiences and writing achievement levels. Like their peers in other English language teacher/translator education programs at Saudi universities, the students in the selected intact class received university instruction in writing English essays of various genres for three terms prior to conducting the study. In the 10-week writing course in which the study was conducted, the students studied how to write 5-paragraph argumentative and classification English essays; this was their first time to study the two essay genres. However, it is worth mentioning that prior to this course, the students had studied how to write compare-and-contrast essays, an essay genre similar to the classification one. All the students were males of Saudi nationality. We focused on male students only to control any potential gender-related differences in students’ responses to genre-based writing instruction, an issue beyond the scope of the present study. All the participants provided informed consent to take part in the study on a voluntary basis after explaining the purpose of the study to them and confirming the confidentiality of their personal data.

### 3.2 The genre-based writing instruction used

As indicated above, the study was conducted in an English writing university course which lasted for 11 academic weeks, two classes of 3 h per week. In the first 5 weeks, the students studied argumentative essay writing, and in the last 5 weeks they studied classification essay writing. The second author was the faculty member who taught the course to the 21 students. In teaching each of the argumentative and classification essays to the students, the following three phases were followed:

- *Phase 1: Getting students to read model texts in the target genre*. This phase lasted for two academic weeks and it aimed at fostering students’ writing knowledge through getting them to analyze the rhetorical features and structure of the target essay genre. In this phase, the students read ten model essays of the target genre. They started by reading four model essays with marked rhetorical and linguistic features so as to guide them in understanding the genre; and then they collaboratively and later independently read the remaining six model essays and identified their the main rhetorical features, and the common phrases and words used in them.
- *Phase 2: Getting students to collaboratively model essay parts in the target genre*. This phase was implemented in the second and third weeks of teaching each genre. In this phase, the students collaboratively wrote essay parts in the classroom, and this was followed by teacher feedback. In the third week, their collaboratively composed essay parts were peer evaluated, and then received teacher feedback on essays parts. The peer feedback activities were guided by using an evaluation rubric for assessing the inclusion of particular rhetorical features in each essay part.
- *Phase 3: Assigning students independent writing tasks in the target genre.* In this phase, which was implemented in the fourth and fifth weeks of teaching each genre, the students were asked to independently write two essays in the target genre as homework assignments, one essay in each week. Then the essays written by all the students in each week were anonymously complied in Word files for teacher feedback and peer evaluation in the classroom. Like the previous phase, peer feedback activities in this one were also guided by the evaluation rubric. In the two phases, the teacher supported the students’ writing learning activities through scaffolding.

### 3.3 Instruments

The study used following the following types of instruments:

#### 3.3.1 Writing tasks

To test students’ writing performance before and after the genre-based instruction, we used a pre-test and a post-test of two obligatory argumentative and classification writing tasks each. The tasks in the two tests were different to avoid the potential influence of topic familiarity on students’ writing performance on the post-test. The writing tasks in the pre-test asked the students to argue for or against the negative influence of social media (the argumentative task), and to talk about the different learning styles: learning by doing things, by reading about things; by listening to people, and talking about things (the classification task). In the post-test, the students were asked to argue for or against having a job while studying at university (the argumentative task), and to talk about the various types of restaurants in Saudi Arabia (the classification task). As noted, the tasks in the two tests do not require specialized knowledge. The time allocated for completing each test was 2 h.

#### 3.3.2 Writing motivation measures

The study used the following four measures to assess the students’ writing motivation before and after instruction:

- *The English Writing Apprehension Scale*. This is a 12-item measure that assesses students’ apprehension of writing and writing evaluation situations. This scale was developed previously by Abdel Latif (2015). The following are examples of the statements included in the scale: “*I usually do my best to avoid writing English essays, I do not like my English essays to be evaluated,* and *I have no fear of my English writing being evaluated*.” A 5-point Likert-type scale (strongly agree, agree, uncertain, disagree, and strongly disagree) is used with the statements. Scores on the scale range from 12 (the minimal score) to 60 (the maximal score). The scale had an Alpha Cronbach reliability coefficient of 0.85.
- *The English Writing Anxiety Scale*. This 12-item scale measures students’ situational feelings or how anxious they feel while performing the writing tasks. The scale is divided into two parts: the items in first part were adapted from Cheng (2004) Second Language Writing Anxiety Inventory and this part asks students to rate their anxious feelings in writing situations, whereas the items in the second part were adapted from Woodrow’s (2011) writing anxiety scale and it asks them to rate how anxious they feel when producing particular text parts. Examples of the items given in the first part: “*I tremble or perspire when I write English essays under time pressure,* and *I feel panicky when I start writing an English essay*”; examples of the items given in the second part: “*How anxious were you when trying to do the following writing activities? Writing sentences without mistakes, and writing a well-organized paragraph*.” A 5-point Likert-type scale (always, often, not sure, seldom and never) is used with the statements in the first part, and another 5-point Likert-type scale is used with the statements in the second one (very anxious, anxious, not sure, non-anxious, and non-anxious at all). Scores on this scale range from 12 (the minimal score) to 60 (the maximal score). The scale had an Alpha Cronbach reliability coefficient of 0.76.
- *The English Writing Self-Efficacy Scale*. This 10-item scale is a modified version from the one used previously in Abdel Latif (2015) study. It measures students’ confidence in writing a text with particular features, or performing task-specific writing skills such as correctly punctuating sentences, organizing sentences, writing sentences with appropriate grammatical structures and vocabulary, and writing essays parts and paragraphs appropriately. Examples of the statements included in the scale: “*When writing English essays, I am able to: correctly punctuate sentences and paragraphs, write an interesting introduction with a good thesis statement, organize sentences into a paragraph so as to clearly express a theme,* and *write an interesting conclusion paragraph*.” A 5-point Likert-type scale (always, often, not sure, seldom and never) is used with the statements. Scores on the scale range from 10 (the minimal score) to 50 (the maximal score). This modified version of the English Writing Self-Efficacy Scale had an Alpha Cronbach reliability coefficient of 0.81.
- *The English Writing Self-concept Scale*. This 12-item measure taps students’ beliefs about and confidence in their general writing abilities and the improvability and learnability of writing. The 12 items in this scale were adapted from previous relevant measures (e.g., Palmquist and Young, 1992; Pajares et al., 2001, 2007; Ehm et al., 2014; Limpo and Alves, 2014). Examples of these items include: “*I believe I was born with the ability to write well in English, my teacher thinks I am a good writer, I cannot improve the quality of my English essays,* and *I write better than other students in my class*.” A 5-point Likert-type scale (always, often, not sure, seldom and never) is used with the statements. Scores on the scale range from 12 (the minimal score) to 60 (the maximal score). This adapted version of this scale had an Alpha Cronbach reliability coefficient of 0.74.

With regard to the validity of the above measures, the scales assessing the students’ English writing apprehension and self-efficacy (the first and third measures) have already been validated in a previous work (see Abdel Latif, 2015), whereas the scales assessing their English writing anxiety and self-concept (the second and fourth ones) were validated through our discussion of how their items match the adopted definitions of the two constructs. In our discussion, we followed the guidelines suggested for validating writing motivation measures (Abdel Latif, 2021). Through this discussion, we concluded that the items of these two scales match our definitions of the two writing motivation constructs.

#### 3.3.3 Post-course evaluation questionnaire

The study also used an open-ended post-course evaluation questionnaire for collecting data about students’ writing learning and motivation experiences after receiving the instructional treatment. The students’ answers to the questionnaire were used to supplement the quantitative data. The rationale for using the open-ended questionnaire rather than interviews is that it minimizes the students’ social desirability, given that they would respond to it anonymously. The questionnaire includes eight open-ended questions which are primarily concerned with the students’ evaluation of the teaching technique used, the perceived strengths and weaknesses of the course, and the potential changes in their writing performance and motivation. The questionnaire was written in Arabic and students were asked to answer the questions in Arabic so as to communicate their perceptions and evaluations more easily. It was also written using Google Forms to enable the students complete it out of the classroom and thus provide as objective answers as possible.

### 3.4 Data collection and analysis procedures

The data collection process was completed through a number of steps. It started with administering the pre-measures (the writing test and the writing apprehension, anxiety, self-efficacy and self-concept scales) to the students. These instruments were administered by the second author during the first week in an additional session that lasted for two and a half hours (30 min for completing the motivation measures and 2 h for completing the writing tasks). Following the provision of the genre-based instruction in 10 weeks, the second author administered the post-measures (the writing test and the writing apprehension, anxiety, self-efficacy and self-concept scales) in another additional 2.5-h session. While completing the pre- and post-tests of writing, the students were not allowed to access any online materials, but they could use printed dictionaries. This was intended to prevent any potential plagiarism attempts from online sources; students’ copying of online materials would not reflect their real English writing levels. Finally, at the end of the last academic week (week 10), the students were asked to complete the open-ended questionnaire on their own out of the classroom. This last procedure was followed to minimize their potential social desirability.

After collecting the data, we collaboratively analyzed it. We started by marking the students’ essays on the pre- and post-tests using Jacobs et al.'s (1981) ESL Composition Profile with which the text is rated in terms of its content (30 points), organization (20 points), vocabulary (20 points), language use (25 points), and mechanics (spelling and punctuation) (5 points); thus totaling 100 points. The essays the students wrote on the pre- and post-tests were co-rated by the second and third authors, and the mean scores for both ratings were calculated. To check the reliability of the essay marking made by each rater, we conducted Cronbach’s alpha reliability analyses of the scores the two raters gave to the five above-mentioned analytic text quality dimensions in all the essays scored. The scores given by the two raters had an average Cronbach’s alpha reliability coefficient of 0.93. The students’ pre- and post-scores on the motivational measures were also counted. Following this, we compared the students’ pre- and post-treatment scores on the writing tasks and psychological measures. Kolmogorov–Smirnov and Shapiro–Wilk tests showed that the data was not normally distributed, and this means non-parametric tests were more suitable for analyzing it. Therefore, we analyzed the data inferentially using the Wilcoxon Signed Ranks Test which is a well-suited non-parametric test. As for the questionnaire data, we independently read the students’ answers to the questions to identify the dominant themes in them. We then had an online meeting for discussing our qualitative data analysis, and for agreeing on the optimal way for presenting the questionnaire data.

## 4 Results

In the following three subsections, we present the results of the data analyses. As will be noted, the presentation of these analyses is guided by the three research questions.

### 4.1 The impact of genre-based instruction on the students’ argumentative and classification essay writing performance

Table 1 shows the means and standard deviations of the students’ analytic scores on the pre- and post-tests of argumentative and classification writing. It can be noted that the students’ scores on all the textual dimensions have increased on the post-test as compared to the pre-test. Compared to the ideational and organizational text aspects, the students’ vocabulary and language use (i.e., language-related text aspects) mean scores on the pre-test were lower (*M* = 8.3 and 9.1, respectively). But given the writing assessment rubric points allocated to both vocabulary and language use (20 and 25 points, respectively), it is noted that the students made better improvements in these two areas compared to the ideational and organizational text aspects on the argumentative tasks, that language use was generally the writing area in which they made the best improvement.

**Table 1:** The means and standard deviations of the students’ writing scores on the pre- and post-tests.

Text quality aspect scores	Argumentative task				Classification task			
	Pre-testing		Post-testing		Pre-testing		Post-testing	
	*M*	SD	*M*	SD	*M*	SD	*M*	SD
Content	15.5	2.8	22.6	3.6	17.7	5.1	24.5	5.2
Organization	9.4	2.4	15.1	1.9	10.4	3.8	16.1	3.7
Vocabulary	8.3	1.6	15.9	2.3	11	3.9	15.9	2.5
Language use	9.1	3.2	18.7	3.4	11	6.1	20	5.1
Mechanics	2.8	0.8	4.2	0.7	3.1	1.1	4.1	0.97
Total score	45.2	10.4	76.7	11.6	54.1	19	80.6	16.9

Meanwhile, the students’ writing mechanics scores considerably improved on the argumentative task (*M* = 2.8 and 4.2 on the pre- and post-tests, respectively) but not that much on the classification one (*M* = 3.1 and 4.1 on the pre- and post-tests, respectively). This might have been caused by the task completion order, given that the students were supposed to complete the argumentative writing task before the classification one. Meanwhile, it is also noted that the students’ pre-test scores on the classification task are higher than those of the argumentative one (*M* = 54.1 and 45.2, respectively), but the differences between the total scores on the two tasks in the post-test decreased (*M* = 80.6 and 76.7, respectively). As implied above, the students’ higher scores on the classification task may have been caused by their previous study of the compare-and-contrast essay type which is similar to the former one.

The Wilcoxon Signed Ranks Test confirmed the significance of these noted differences. Table 2 gives the results of the Wilcoxon Signed Ranks Test analyses of the students’ writing scores on the pre- and post-tests. As the table show, all these mean differences are significant with the exception of the mechanics mean scores on the classification task. Overall, these significant differences indicate that genre-based instruction helped the students improve their English argumentative and classification essay writing in terms of text content, organization, vocabulary, and language use. As a result, genre-based instruction also helped in significantly improving the students’ total essay scores on both task types.

**Table 2:** The Wilcoxon Signed Ranks Test analyses of the students’ writing scores on the pre- and post-tests.

Text quality aspect scores	Task type	Mean ranks		Sum of ranks		Z	*p*-value
		N. ranks	P. ranks	N. ranks	P. ranks		
Content	Argumentative task	2.5	10.9	2.5	207.5	3.832	0.000
	Classification task	7.2	10.3	14.5	175.5	3.245	0.001
Organization	Argumentative task	0.0	10.5	0.0	210	3.931	0.000
	Classification task	8.7	17.5	11.2	213.5	3.410	0.001
Vocabulary	Argumentative task	0.0	11	0.0	231	4.025	0.000
	Classification task	10	10.5	20	190	3.178	0.001
Language use	Argumentative task	0.0	11	0.0	231	4.018	0.000
	Classification task	8.5	10.7	17	193	3.287	0.001
Mechanics	Argumentative task	0.0	9.5	0.0	171	3.810	0.000
	Classification task	10.7	10.4	43	167	2.380	0.017
Total scores	Argumentative task	0.0	11	0.0	231	4.015	0.000
	Classification task	6.7	10.9	13.5	196.5	3.416	0.001

N. ranks, negative ranks; P. ranks, negative ranks.

### 4.2 The impact of genre-based instruction on the students’ writing motivation

Table 3 provides the means and standard deviations of the students’ scores on the pre- and post-measures of writing motivation. As noted, there are improvements in the four writing motivations dimensions. The largest differences between the students’ mean scores on the pre- and post-measures of writing motivation are in those of writing anxiety and self-concept, respectively. Meanwhile, the students attained better improvements in fostering their writing self-efficacy than in alleviating their writing apprehension (*M* = 26.7 versus 40.9 out of 50 for the writing self-efficacy measure, and 40.9 versus 26.1 out of 60 for the writing apprehension measure).

**Table 3:** The means and standard deviations of the students’ scores on the pre- and post-measures of writing motivation.

Writing motivation variables	Pre-testing		Post-testing	
	*M*	SD	*M*	SD
Writing apprehension	40.9	14.5	26.1	11.8
Writing anxiety	41.4	14.1	21.9	10.2
Writing self-efficacy	26.7	13.6	40.9	9.4
Writing self-concept	30.5	16.4	46.1	9.7

The significance of these noted differences was also confirmed by the Wilcoxon Signed Ranks Test. Table 4 gives the results of the Wilcoxon Signed Ranks Test analyses of the students’ scores on the pre- and post measures of writing motivation. The table shows that the differences between the students’ mean scores on the pre- and post-measures of the four writing motivation dimensions are all statistically significant. This indicates that the genre-based instruction has led to decreasing the students’ writing apprehension and anxiety and also enhancing their writing self-efficacy and self-concept.

**Table 4:** Results of the Wilcoxon Signed Ranks Test analyses of the students’ scores on the pre- and post measures of writing motivation.

Writing motivation variables	Mean ranks		Sum of ranks		Z	*p*-value
	N. ranks:	P. ranks:	N. ranks:	P. ranks:		
Writing apprehension	11.3	7.1	181.5	28.50	−2.857-b	0.004
Writing anxiety	11.2	3.3	180	10	−3.422-b	0.001
Writing self-efficacy	4.4	12.5	22	188	−3.101-c	0.002
Writing self-concept	5.7	12.2	23	208	−3.217-c	0.001

N. ranks, negative ranks; P. ranks, negative ranks.

### 4.3 The students’ evaluation of genre-based writing instruction and perceptions of their writing motivation development

The students’ answers to the open-ended questionnaire questions supported the above quantitative data about the gains in their English writing performance and writing motivation. Nineteen of the 21 students reported very positive views on the genre-based instruction they received. These students found genre-based writing instruction a completely different teaching technique from the ways English writing were taught to them previously. They particularly liked the idea of studying multiple essay models and analyzing their structures. This can be noted in the following two exemplary answers:−I liked this teaching method very much and I found it interesting and useful. It requires critical thinking and analysis, and differs completely from the teaching methods in our previous essay writing courses. It depends on our collaborative analysis of the models to understand the essay organization, vocabulary and the language.

- *In my opinion, this is a very good teaching method as we have seen and analyzed many essay models. I found a clear focus on developing our writing skills through guiding and motivating us to analyze the model essays and notice their steps and main language elements, and also to improve our writing skills in presenting ideas and arguments logically. The classroom activities helped us greatly in understanding how to write essays in a better way. For me, this is a completely new method.*

In their answers to another questionnaire question related to the perceived benefits of genre-based instruction, they students mentioned a number of positive aspects. Collectively, these include: modeling texts or having a model to guide one’s writing, composing the text following certain steps, developing vocabulary and structures related to the target essay type, becoming more motivated writers, having a more positive attitude toward collaborative writing and classroom activities, understanding the process of learning how to learn writing, and recognizing the importance of reading for writing. In the two answers below, the students refer to some of these benefits:

- *Yes, I benefited how to write different essay types based on finding and reading model essays and analyzing their grammar, connectors, and vocabulary. I also learned how to write a well-organized essay by using appropriate structures and arguments, and related vocabulary. My confidence in my writing has greatly increased.*
- *I learned many things in this course. First, collaborative work is very important in learning writing. Second, analyzing model essays is essential to know the vocabulary and grammar I’m supposed to use. I also understood how to organize my essays and to model an essay I have read.*

On the other hand, four students congruently reported that genre-based instruction is time-consuming and that it requires many efforts from them as students. They generally viewed it is inappropriate for the students with low writing motivation or poor language proficiency. One student expressed these concerns as follows:

- Honestly, I did not like this method because it requires many things such as time and effort. Yes, it is a different teaching method but it requires me to do a lot in the classroom.

The above answer suggests that some students in this context may not be used to learner-centered teaching.

With regard to the students’ perceived improvement in English writing, the vast majority of them reported experiencing writing development perceptions as a result of receiving genre-based instruction. For some students, such perceived improvement was associated with the higher essay scores and more positive teacher feedback they received during the course. For other students, they had improved perceptions of their writing ability as a result of developing particular writing skill dimensions such as: having fluency with generating ideas and putting them into language, writing more text quantity easily, and organizing the text more effectively. The following exemplary answers indicate some of these factors:

- *Yes, my writing has improved. This is indicated by the higher scores I got on my essays this term.*
- *Sure. Now I can find ideas quickly and vocabulary easily, and I no longer think in Arabic when writing.*
- *Yes, I can write more easily and in a faster way now. In the previous terms, I used to take a long time to write an essay of 100 or 150 words, but now I can write 500 words in about 3 h.*
- *I feel my English writing has improved a lot as I learned how to organize my ideas and opinions in a better way, and how to support my ideas.*

With such perceived improvements in their writing performance, many students mentioned in their answers to the final interview question called for using genre-based instruction in the future writing courses they would attend.

## 5 Discussion and conclusion

As indicated above, the use of genre-based instruction has resulted in improving the students’ English argumentative and classification writing performance in terms of text content, organization, vocabulary, language use, and– to a less extent– mechanics. Relatively better improvements were particularly noted in the students’ language use and vocabulary, respectively. These writing performance improvements have been accompanied by enhancement of the students’ writing motivation. Specifically, the students’ English writing self-efficacy and self-concept increased whereas their English writing apprehension and anxiety decreased. The students’ answers to the open-ended questionnaire supported the results of the quantitative data. The students particularly liked some features in genre-based writing instruction such as studying multiple model texts and understanding the vocabulary used in the target genre, and they perceived some improvements in their writing performance such as becoming more aware of genre rhetorical features, and developing a better writing fluency level.

These results concur with previous research findings that genre-based instruction fosters students’ writing ability (e.g., Martínez Esteban and Roca de Larios, 2010; Yasuda, 2011; Van de Poel and Gasiorek, 2012; Han and Hiver, 2018; Luquin and Mayo, 2021). Besides, these results emphasize the view that genre-based instruction enhances students’ linguistic and communicative abilities (e.g., Leki et al., 2008; Paltridge, 2013). Overall, the present results support the conclusion that when students develop an expected level of language awareness and writing competence, they become more motivated to write (e.g., Cheng et al., 1999; Pajares and Valiante, 2001; Hertz-Lazarowitz and Bar-Natan, 2002; Abdel Latif, 2015, 2019; Limpo and Alves, 2017; Torres et al., 2020). Our results are also consistent with those of the few published studies indicating that genre-based instruction enhances students’ writing motivation (e.g., Yasuda, 2011; Van de Poel and Gasiorek, 2012; Han and Hiver, 2018). The study emphasizes the importance of instructional practices in shaping students’ writing motivation (e.g., Lo and Hyland, 2007; Behizadeh and Engelhard, 2014).

Overall, the above results imply that some particular writing instruction characteristics are conducive to developing L2 students’ writing performance and nurturing their writing motivation. These characteristics include: exposing students to multiple model texts, familiarizing them with the common vocabulary and organizational patterns of the target text genre, engaging them in collaborative writing activities, raising their awareness of effective writing processes, and developing their ideational and linguistic fluency. As implied, teacher scaffolding and learner active role are key factors for genre-based writing instruction to be effective. Due to its potential benefits, we suggest incorporating genre-based instruction– or at least some of its above-mentioned features– into English writing courses in the Saudi university context in particular, and perhaps into other similar L2 writing learning environments. Once students reach the target level(s) in genre and language awareness, teachers can depend more on writing process instruction. This generally means that L2 writing instruction should be tailored to meet students’ linguistic and strategic needs in order to positively influence their writing motivation (Abdel Latif, 2015, 2019, 2021).

While the present study has revealed important insights into the role of instruction in students’ writing motivation, other relevant issues remain to be addressed in future research. There is a need for experimenting genre-based writing instruction in other educational stages and contexts to explore students’ potential performance and motivation responses to it. Given that the present study used the one-group pre-post-test design, researchers interested in employing genre-based writing instruction in their futures studies may experiment it using other research designs. Besides, we need to examine the potential impact of other instructional techniques on students’ writing motivation. Technology-supported instruction and feedback treatment types particularly deserve further research attention. In previous writing instructional research, it is generally noted that motivation has been addressed peripherally (see Abdel Latif, 2021). In future writing instruction studies, researchers need to give writing motivation constructs a prominent place, and to cover a wider range of them. Such research could help us have a clearer idea about the relative effects of various instructional types on students’ writing motivation dimensions.

## Author’s note

Muhammad M. M. Abdel Latif is an associate professor of TESOL at the Faculty of Graduate Studies of Education. Cairo University, Egypt. He has published research papers in more than 15 internationally well-known and ranked journals, including: *Applied Linguistics, Assessing Writing, Canadian Modern Language Review, ELT Journal, Journal of Computer Assisted Learning, ReCALL,* and *System.* He is also the author of *Writing Motivation Research, Measurement & Pedagogy* published by Routledge.

Talal Musaed Alghizzi is an associate professor of applied linguistics at Imam Mohammad Ibn Saud Islamic University (IMSIU), Saudi Arabia. He completed his PhD at the University College Cork, Republic of Ireland. He has published in WoS- and Scopus-indexed journals.

Tahani Munahi Alshahrani is an associate professor of applied linguistics at Imam Mohammad Ibn Saud Islamic University (IMSIU), Saudi Arabia. She has published in WoS- and Scopus-indexed journals.

## Data availability statement

The raw data supporting the conclusions of this article will be made available by the authors, without undue reservation.

## Ethics statement

The studies involving humans were approved by The Deanship of Scientific Research at Imam Mohammad Ibn Saud Islamic University. The studies were conducted in accordance with the local legislation and institutional requirements. The participants provided their written informed consent to participate in this study.

## Author contributions

MA: Conceptualization, Methodology, Project administration, Writing – original draft. TAlg: Conceptualization, Data curation, Formal analysis, Investigation, Validation, Writing – review & editing. TAls: Conceptualization, Data curation, Funding acquisition, Methodology, Validation, Writing – review & editing.
