# Correction: The impact of genre-based instruction on Saudi university students' English writing performance and motivation: a mixed-method study

**DOI:** 10.3389/fpsyg.2025.1682334

**Published:** 2025-09-05

**Authors:** Muhammad M. M. Abdel Latif, Talal Musaed Alghizzi, Tahani Munahi Alshahrani

**Affiliations:** ^1^Faculty of Graduate Studies of Education, Cairo University, Giza, Egypt; ^2^College of Languages and Translation, Imam Mohammad Ibn Saud Islamic University (IMISU), Riyadh, Saudi Arabia

**Keywords:** genre-based instruction, writing motivation, writing pedagogy, writing psychology, writing performance, genre-based writing instruction

In the published article, there was an error in the author's correspondence details. Muhammad M. M. Abdel Latif, email: mmmabd@cu.edu.eg, was erroneously listed as the corresponding author. The correct corresponding author is Talal Musaed Alghizzi, email: tmalghizzi@imamu.edu.sa.

In the published article, there was also an error in the Funding statement. The grant number was erroneously listed as “IMSIU-RG23153.” The correct grant number is “IMSIU-DDRSP2504.”

The original article has been updated.

